# Polyphenol Composition by HPLC-DAD-(ESI-)MS/MS and Bioactivities of Extracts from Grape Agri-Food Wastes

**DOI:** 10.3390/molecules28217368

**Published:** 2023-10-31

**Authors:** Jonata M. Ueda, Karoline Ribeiro Griebler, Tiane C. Finimundy, Daniele B. Rodrigues, Lavínia Veríssimo, Tânia C. S. P. Pires, João Gonçalves, Isabel P. Fernandes, Eliana Pereira, Lillian Barros, Sandrina A. Heleno, Ricardo C. Calhelha

**Affiliations:** 1 Centro de Investigação de Montanha (CIMO), Instituto Politécnico de Bragança, Campus de Santa Apolónia, 5300-253 Bragança, Portugal; jonata.ueda@ipb.pt (J.M.U.); karolinegriebler@alunos.utfpr.edu.br (K.R.G.); tiane@ipb.pt (T.C.F.); daniele@ipb.pt (D.B.R.); laviniasverissimo@gmail.com (L.V.); tania.pires@ipb.pt (T.C.S.P.P.); eliana@ipb.pt (E.P.); lillian@ipb.pt (L.B.); calhelha@ipb.pt (R.C.C.); 2 Laboratório Associado para a Sustentabilidade e Tecnologia em Regiões de Montanha (SusTEC), Instituto Politécnico de Bragança, Alameda Santa Apolónia, 5300-253 Bragança, Portugal; 3 Tree Flowers Solution, Lda, Edificio Brigantia Ecopark, Av. Cidade de Léon, 5300-358 Bragança, Portugal; joaogoncalves@treeflowerssolutions.com (J.G.); isabelfernandes@treeflowerssolutions.com (I.P.F.)

**Keywords:** grape marc, by-products, phenolic compounds, antioxidant, antimicrobial

## Abstract

Background: Grape agri-food wastes, such as skin, seeds, and other discarded by-products, contain phytochemical compounds that offer potential health benefits. Methods: This study aimed to investigate the polyphenol composition and bioactivities of different extracts obtained from grape marc and seeds, with the goal of exploring their potential for application as natural food additives. Results: Regardless of the extraction method used (dynamic maceration, ultrasound-assisted extraction (UAE), and microwave-assisted extraction (MAE)), all extracts exhibited relatively high concentrations of phenolic compounds. The chemical characterization of the extracts revealed the presence of specific compounds and chemical groups associated with each extraction methodology. Moreover, the extracts displayed satisfactory antioxidant activities, especially in inhibiting lipoperoxidation as assessed by the TBARS assay. Additionally, the extracts demonstrated effective inhibition against different strains of bacteria and fungi known as food contaminants. Taken together, these findings indicate that those extracts have the potential to be tested as natural antioxidants and preservatives with sustainable origins in food and beverage systems. Among the extraction methods evaluated, traditional maceration and UAE provided extracts with the highest antioxidant and antimicrobial activities. Conclusions: Our results suggest the opportunity to explore grape marc and seeds discarded by the winery industry in Portugal as natural sources of bioactive compounds, which could be employed as functional food ingredients or technological additives. The valorization of grape biowastes offers a promising strategy to reduce waste and harness their potential health benefits.

## 1. Introduction

Wine, produced mainly from *Vitis vinifera*, is one of the most valuable and consumed alcoholic beverages throughout the world, with an annual production of approximately 27 billion litres [1,2]. Europe is recognised for its high-quality wines, and in this scenario, Portugal stands out for its traditional and expressive production of wines with Protected Designation of Origin (PDO) and Geographical Indication (PGI) labels, highlighting the distinctive wine production across the 14 different regions of the country [3,4]. To exemplify it, in a recent competition in Portugal, over 1300 Portuguese wines were sensorially evaluated by 151 experts and most received scores ranging between 80 and 90 on a 0–100 scale, corroborating the level of quality and diversity of the wines produced [5].

Nonetheless, about 75 million tons of grape by-products and bioresidues are generated annually from industrial grape processing worldwide, with grape pomace and grape marc representing the largest share of these residues, exceeding 10 million tons/year [6]. The valorization of the solid residue from the agri-food sector including wineries can mitigate potential environmental impacts and foster the development of high-added value products, which benefit the entire production chain and align with a sustainable perspective [7]. Commonly, grape bioresidues have been destined for animal feed, composting, seed oil extraction, and the production of alcoholic beverages [6].

Grape bioresidues are widely studied for their putative health benefits, mainly related to their composition of phytochemicals recognised as bioactive, especially phenolic compounds. These compounds are plant secondary metabolites, and, as such, are differently produced in response to environmental stimuli, edaphoclimatic conditions, and genetic factors. Grape polyphenols are mainly represented by anthocyanins that impart their red to purple colors, as well as non-anthocyanin compounds comprising, for instance, tannins and stilbenes such as resveratrol [7]. Present in large amounts in winery bioresidues, grape skin and seeds present generally higher concentrations of phenolic compounds than its pulp [8,9,10]. Among the benefits associated with phenolic compounds from grape pulp and bioresidues, their cardioprotective, hepatoprotective, antidiabetic, and anticancer effects are highlighted [8,9,10]. Besides their beneficial properties in relation to health, phenolic compounds may play interesting roles as technological or functional food ingredients. De Francesco et al. (2020), for instance, reported that the extracts of green tea and grape seeds, rich in condensed tannins, efficiently extended the shelf-life of beers upon incorporation into this product [11]. The antioxidant and antimicrobial properties of polyphenols have been associated with these findings [12].

Phenolic compounds for industrial applications can be obtained by synthesis, microbial production, and extraction from several matrices. Emerging extraction methods such as those based on ultrasound and microwave technologies have been gaining space at academic and industrial levels, and been used to recover natural molecules as they are considered to be more efficient than traditional methods [7]. Nonetheless, it is necessary first to evaluate the quality of the extracts obtained, for instance in their composition of phytochemicals and bioactivities, to move to more focused and necessary technological development and incorporation in food and beverage prototypes. For extracts from plant residues, the polyphenol composition may vary depending not only on the several conditions aforementioned, but also on those related to the processing that originates the residue and to the extraction of such compounds.

Building on the above, this work aims at evaluating the polyphenol composition and antioxidant and antimicrobial activities of different extracts from winery bioresidues, namely, grape seeds and marc obtained in Portugal, where these residues are particularly representative, to explore their potential applications as natural preservatives, envisaging their valorization. Despite previous research on winery wastes from Portugal, the present study simultaneously evaluates extracts from different extraction protocols performed side-by-side, including two innovative methods (UAE and MAE) and their impacts on polyphenol composition (LC-DAD-MS), and antioxidant (biochemical and chemical-based assays) and antimicrobial activities. The present study includes the assessment of both bacteriostatic and bactericidal concentrations against several food-borne pathogens. This laboratorial screening is relevant to assessing the quality of the extracts obtained, because it provides insights into the potential use of these sustainable polyphenol-rich extracts as natural preservatives in food and beverage systems.

### 1.1. Polyphenol Composition

**Figure 1:**
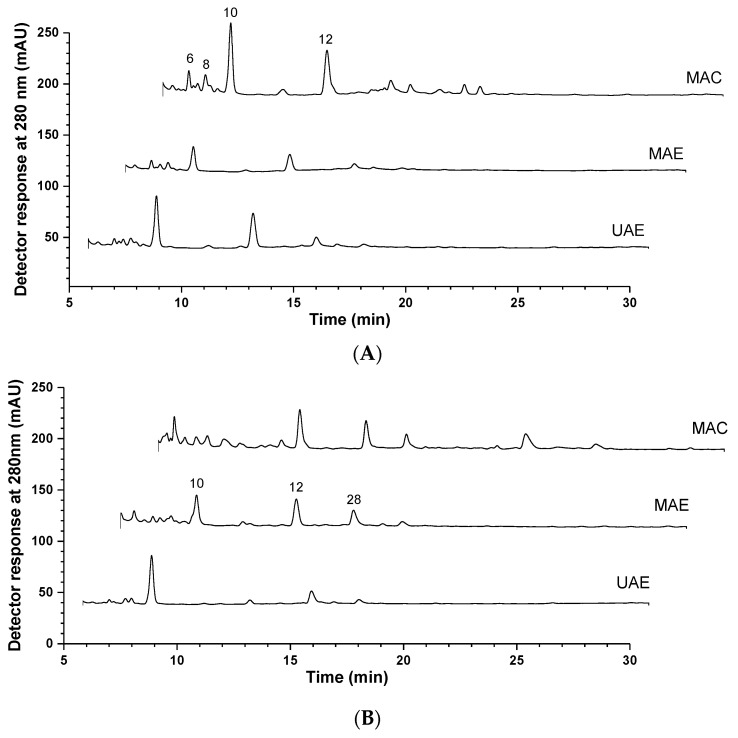
HPLC-DAD phenolic profile of extracts from *V. vinifera* seeds (**A**) and marc (**B**), obtained by stirring maceration (MAC), ultrasound-assisted extraction (UAE), and microwave-assisted extraction (MAE) processes. Chromatograms were processed at 280, 320 and 370 nm, while only representative chromatograms extracts recorded at 280 nm are shown herein. Peak identification and quantification are presented in Table 1.

The identification and quantification of phenolic compounds in *V. vinifera* winery residues are presented in Table 1 and Figure 1. Twenty-nine phenolic compounds were tentatively identified in the extracts prepared from the grape seeds and marc by using three extraction methods (dynamic maceration, ultrasound-assisted and microwave-assisted extraction): four phenolic acids (hydroxybenzoic acid derivatives), two stilbenes (resveratrol derivatives), nineteen flavan-3-ols (catechin derivatives, proanthocyanidins, and their isomers), three flavones (apigenin, luteolin, and syringetin derivatives), one hydrolyzable tannin (galloyl derivative), and five flavonols (quercetin and kaempferol derivatives). Peaks **1, 2, 4, 21** and **26** were positively identified as gallic acid, resveratrol, protocatechuic acid, (+)-catechin and (−)-epicatechin, respectively, by comparison with commercial standards and considering their retention time, and mass and UV-Vis spectra. The stilbene of peak **3** ([M−H]^−^ at *m*/*z* 389) presented a fragment ion at *m*/*z* 227, equivalent to resveratrol after releasing a hexosyl unit (−162 u); therefore, this compound was tentatively identified as resveratrol-3-*O*-glucoside [13]. Regarding the phenolic acid derivatives, peak **20** ([M−H]^−^ at *m*/*z* 331) showed a loss of 162 u in the MS^2^ spectra yielding an ion at *m*/*z* 169, corresponding to the gallic acid molecule. Therefore, it was identified as galloyl glucose. The only hydrolysable tannin was identified in seed extracts; the UV-Vis and mass spectra characteristics of peak **11** ([M−H]^−^ at *m*/*z* 937) coincided with those of a trigalloyl-HHDP-glucoside, previously described by Passos et al. (2007) [14]. In the family of the flavonoid compounds, peaks **5** ([M−H]^−^ at *m*/*z* 593), **13** ([M−H]^−^ at *m*/*z* 609), **14** ([M−H]^−^ at *m*/*z* 593), **16** ([M−H]^−^ at *m*/*z* 447), and **18** ([M−H]^−^ at *m*/*z* 463) were tentatively identified by comparing their chromatographic and spectral characteristics with those described in the literature, being therefore assigned as syringetin-3-*O*-hexoside [15], quercetin-3-*O*-glucoronide [16], and luteolin-glucuronide [17], respectively. Finally, the primary group identified in these samples were condensed tannins formed by the condensation of flavan-3-ols. Peak **25** ([M−H]^−^ at *m*/*z* 577) presented MS^2^ fragments at *m*/*z* 451 (−126 u), 425 (−152 u), and 407 (−152–18 u), and *m*/*z* 289 and 287, coherent with the loss of two catechin units, being therefore tentatively identified as B-type procyanidin dimer. Peak **8**, with the same precursor ion ([M−H]^−^ at *m*/*z* 865), exhibited similar mass spectra parameters to the B-type procyanidin trimer elucidated in grape juice [18]. Peak **24** ([M−H]^−^ at *m*/*z* 863) showed the same fragmentation pattern as peak **8**, except for having 2 u less than the formers. Therefore, this compound was identified as an A-type-like procyanidin trimer. The same case occurred in peak **23** ([M−H]^−^ at *m*/*z* 867), which is a procyanidin trimer C. Peaks **6** ([M−H]^−^ at *m*/*z* 1169), **7**, and **29** ([M−H]^−^ at *m*/*z* 1017) revealed the presence of a galloyl group in their composition. According to a study carried out with grape pomace, this compound was tentatively identified with di- and mono-galloylated type B-linked procyanidin trimer. Peaks **9 and 12** ([M−H]^−^ at *m*/*z* 1153) were assigned as B-type procyanidin tetramers. **Peaks 22** ([M−H]^−^ at *m*/*z* 1137) and **28** ([M−H]^−^ at *m*/*z* 1121) were tentatively identified as proanthocyanidin tetramers, B-type and A-type linked, respectively. Finally, peaks **10,** and **27** ([M−H]^−^ at *m*/*z* 1439) were assigned as procyanidin pentamer.

Overall, the three extraction methodologies provided extracts with similar polyphenol profiles among those prepared from the same sample (Figure 1). Similarities in the chromatographic profile can also be noticed between the seed and marc extracts, as overall, they shared the same major compounds. In fact, the most abundant compounds in the extracts analyzed were isomers of procyanidin pentamers and tetramers, in line with Ruberto et al. (2007) [19]. Within the grape seed extracts, the content of procyanidin pentamer (peak 10) ranged from 7.42 ± 0.21 mg/g in the MAC extract to 2.99 ± 0.05 mg/g in the MAE one, accounting alone for 22.8% to 38.4% of the total phenolic compounds of seed extracts. The same compound was the most prevalent in UAE (4.3 ± 0.1 mg/g) and MAE (4.22 ± 0.04 mg/g) extracts of grape marc, whereas the isomer II of procyanidin tetramer (peak 12) was the major compound in MAC extract (4.21 ± 0.04 mg/g). Those individual compounds represented up to 37% of total polyphenols in the respective marc extracts.

As a polyphenol class, the condensed tannins (procyanidins) comprised the largest proportion of phenolic compounds for both grape seed and marc residues, regardless of the extraction procedure employed. The relative percentage of this class ranged from 72 to 74% and from 84 to 88% in seeds and marc extracts, respectively. On the other hand, the stilbenes, represented by the resveratrol (peak 2) and resveratrol-*O*-hexoside (peak 3), were detected only in seed extracts (~1 mg/g extract dw). Moreover, although the absolute presence of total flavonoids was similar between seed and marc extracts (1.3 ± 0.1 to 3.6 ± 0.3 and 1.1 ± 0.02 to 3.3 ± 0.1 mg/g dw, respectively), only the former group presented glycosides of flavones and flavonols.

From a perspective of total compound recovery, grape seeds presented slightly higher levels of total phenolic content than the marc, which ranged from 13.1 ± 0.5 to 27 ± 1 and from 11.6 ± 0.2 to 21.6 ± 0.4, respectively, depending on the extraction procedure employed (Table 1). Additionally, when considering the different extraction procedures, extracts obtained with MAC showed the best yields of phenolic compounds for both samples, and these did not differ from those obtained via MAE when applied to grape marc. For grape seeds, the total phenolic content of MAC extract (27 mg/g dw, *p* < 0.05) was twice those of UAE (15 mg/g dw) and MAE (13.1 mg/g dw) extracts, with no difference between them (*p* < 0.05). In this sample, MAC consistently extracted the highest content of all polyphenolic classes. However, for grape marc, extracts obtained with MAC and MAE showed similar contents of total phenolic compounds (21.6 and 20.3 mg/g dw, respectively), two times the polyphenol content of the extract obtained by using UAE (11.6 mg/g dw). Within the extracts of this sample, the MAC one also presented the highest contents of total condensed tannins and total flavonoids, which did not differ from the MAE extract, which surpassed the former in terms of the content of phenolic acids. Conversely, for seeds, MAE extracts presented overall lower values of total polyphenols and their classes in contrast to MAC. Overall, the extracts obtained with UAE presented the lowest amounts of polyphenols. A study carried out with grape seeds from the North of Portugal, obtained through hydromethanolic extraction, identified six flavonols and 13 flavan-3-ols, mostly catechins and epicatechins, in line with our findings [20]. Compared to skins and mixtures, they also reported that seeds had a higher concentration of phenolic compounds (10.2 mg/g of extract), a content lower than the one found in the present study [20].

Although microwave-assisted extraction was the only method performed with ultrapure water in contrast to 80% EtOH, the only phenolic compound that showed differences in relation to the extraction solvent was quercetin-3-*O*-glucoside, absent only in the MAE extract of grape seeds. No such differences were observed for the grape marc extracts. When all the above results are taken together, the extraction methodology seems to have a greater influence on the phenolic composition than the solvent used, although we cannot separate their contributions.

It is important to note that in this study, different extraction protocols were employed, each one performed according to their established conditions within our laboratory framework. While this setup indeed presents challenges in drawing direct comparisons between the results, it was employed to gain insights into the intrinsic potential and overall quality of the extracts generated from each method. The aim of the study was to evaluate the potentials of grape marc and seed extracts obtained with these protocols to be used as natural food additives, as an initial screening. As such, in this case, it was more relevant to us to assess the overall quality of the extract obtained in terms of composition and bioactivity, rather than to directly compare the extraction efficiency of the three methods under various conditions. In other words, the focus primarily centred on assessing the outcome of the extraction technique, rather than separating the individual effects of the technology, operation, or solvent to evaluate the extraction efficiency per se. As this study progresses towards optimization and scaling-up, it is anticipated that we will achieve direct comparisons of specific parameters.

### 1.2. Antioxidant Activity

For antioxidant capacity, two in vitro chemical assays (DPPH and reducing power) and one biochemical assay (TBARS) were performed (Table 2). The reducing power measures the conversion of the Fe^3+^/ferricyanide complex to the Fe^2+^/ferrous form, while DPPH measures the decreased absorption at 515 nm of the resonating DPPH^●^ radical after its stabilization is achieved with radical scavengers. Finally, the TBARS assay estimates the inhibition of lipid peroxidation in pig brain tissue using antioxidants [21]. The lower the concentration of extract needed to inhibit the oxidative process, reduce the metallic ions, or scavenge the radical, the greater the antioxidant activity.

In the TBARS assay, the UAE extract of grape marc showed the best results (0.023 mg/mL) among all extracts evaluated, while for seeds the highest antioxidant activity was observed for the MAC extract. In this study, no correlation was found between the total phenolic content of the extracts and the antioxidant activity for either sample, suggesting that the synergism between the different phenolic components present in grape marc may be responsible for the observed effects. However, when compared to the phenolic profiles (Table 1), the reducing power and DPPH assays showed results that associate the concentration of total phenolics with the antioxidant activity. The reducing power assay of grape seeds was able to show the best antioxidant activity for maceration extraction (0.097 mg/mL), which also had the highest concentration of phenolic compounds among the different extractions methods (Table 1). Therefore, chemical assays (DPPH and reducing power) provided results that were more consistent with the phenolic composition of different extracts of grape marc and seeds. On the other hand, the biochemical assay (TBARS) showed the best results among the different antioxidant activity assays, suggesting the involvement of other biochemical reactions in the antioxidant activity of the extracts. The discrepancies in TBARS assay results may also be attributed to potential overestimated oxidation, due to the presence of some pigments that show absorbance at 532 nm [22].

For chemical assays, it is evident that phenolic compounds (as shown in Table 1) exert a more pronounced influence on the antioxidant activity results, compared to the biochemical test (TBARS). For DPPH, the assay indicated that phenolic acids played a significant role in the observed results for both grape marc and seeds. However, for reducing power, there were no apparent correlations between the phenolic profile and antioxidant activity, except for stilbenes, which were exclusively identified and quantified in grape seeds.

Regarding the biochemical assay, no similarity was found between the phenolic composition and antioxidant activity. The TBARS assay may not be as suitable for antioxidant analysis, since the sugar and aldehyde contents in both grape marc and seeds can potentially interfere with the absorbance and, consequently, affect with the assessment of lipid oxidation [23,24,25].

The disparities between the results highlight the difficulty of comparing the different antioxidant activity assays, as suggested by the mechanisms of action of each assay. Other authors have also reported the lack of correlation between different assays, as observed in studies involving spice extracts [26] and essential oils [27].

Although DPPH radical-scavenging and ferric reducing antioxidant power (FRAP) assays are based on estimating the total contents of reductants through the removal of the hydrogen atom from antioxidants, the TBARS assay works through brain tissue oxidation, and many factors can influence the result, including the catalyst used, temperature, and analysis time, all of which involve lipid peroxidation [21,28,29].

### 1.3. Antimicrobial Activity

Table 3 presents the antibacterial and antifungal activity of grape by-product extracts against Gram-positive (*Enterobacter cloacae*, *Escherichia coli*, *Pseudomonas aeruginosa*, *Salmonella enterocolitica*, and *Yersinia enterocolitica*) and Gram-negative bacteria (*Bacillus cereus*, *Listeria monocytogenes*, and *Staphylococcus aureus*), as well as two fungi (*Aspergillus brasiliensis* and *Aspergillus fumigatus*), considered contaminants in food products.

Among grape marc extracts, the MAE extract did not present antibacterial activity at the maximum concentration tested (10 mg/mL) for any of the Gram-positive bacteria. However, for Gram-negative bacteria, the extract inhibited *L. monocytogenes* and *S. aureus* at a concentration of 10 mg/mL. Extracts obtained through UAE and MAC were able to inhibit, at a concentration of 10 mg/mL, the Gram-positive bacteria *E. cloacae*, *E. coli*, and *P. aeruginosa.*

For grape seeds, the extract obtained by maceration showed the best results, with antibacterial activity against both Gram-positive (*S. enterocolitica*) and Gram-negative bacteria (*B. cereus*, *L. monocytogenes*, and *S. aureus*) at a concentration of 10 mg/mL. The UAE extract inhibited the growth of *S. aureus* at 10 mg/mL, while the MAE one did not show any antibacterial activity at the maximum concentration tested (10 mg/mL).

Although some grape seed and marc extracts did not show inhibitory activity at the maximum concentration tested, it is possible that a reduction in the microbial load may still occur when applied to foods [30]. On the other hand, essential oils extracted from grape seeds have been shown to have notorious antibacterial activity, attributed to the presence of terpenes in the oils [31].

Although gallic acid is effective in inhibiting *E. coli* and *Salmonella* spp. [32] and is present in all seed and grape marc extracts (Table 1), the results presented in Table 3 show that, in some of the extracts, no inhibition was observed at the maximum concentration tested. This suggests a potential antagonistic effect between the components present in the extract, and the maximum tested concentration (10 mg/mL) may be insufficient for practical application in food as a natural preservative.

These antifungal findings corroborate with the results obtained by other authors [33], in which grape by-product extracts were found to be effective against *Candida* spp. Among the compounds present, flavan-3-ols and flavonols (quercetin-3-*O*-glucuronide) are found in both grape seed and marc extracts (Table 1). Although phenolic compounds, in general, are associated with the bioactivity of plant extracts, it is worth noting that stilbenes, present in grape marc (Table 1), exhibit a greater antimicrobial effect in comparison to phenolic acids and flavonoids, even at lower concentrations in the extract [34].

## 2. Material and Methods

### 2.1. Samples, Standards and Reagents

Samples of *Vitis vinifera* by-products (seeds and marc) were obtained from Destildouro—Destilações do Douro (Portugal). Grape marc refers to the solid residue from winemaking, after grapes have been fermented and pressed, encompassing grape seeds and skin. All samples were reduced to a fine powder (20 mesh) that was stored at room temperature (~25 °C) under vacuum and protected from light until further analysis. HPLC-grade methanol, acetonitrile, and ethanol (EtOH, 99.9%) were provided by Fisher Scientific (Leicestershire, UK). Ultrapure water (H_2_O) was obtained through a water purification system (TGI Pure Water Systems, Greenville, SC, USA). Formic acid was purchased from Prolabo (VWR International, Rosny-sous-Bois, France). Phenolic compound standards (gallic acid, resveratrol, protocatechuic acid, apigenin-7-*O*-glucoside, (+)-catechin, quercetin-3-*O*-rutinoside, kaempferol-3-*O*-glucoside, quercetin-3-*O*-glucoside, luteolin-7-*O*-glucoside, and apigenin-7-*O*-glucoside) were acquired from Extrasynthese (Genay, France). Trolox, Tris, ascorbic acid, trichloroacetic acid, ellipticine, and dimethyl sulfoxide were obtained from Sigma-Aldrich (St. Louis, MI, USA), and thiobarbituric acid, calcium chloride, magnesium chloride, *p*-iodonitrotetrazolium chloride (INT) sodium sulfate and tryptone soy broth were acquired from Panreac Applichem (Barcelona, Spain). 2,2-diphenyl-1-picrylhydrazyl (DPPH) was obtained from Alfa Aesar (Ward Hill, MA, USA). Iron (II) sulfate was acquired from ACROS Organics (Geel, Belgium). Anovine blood sample was obtained from healthy animals donated by the School of Agriculture, Bragança, Portugal, and porcine (*Sus scrofa*) brain material was obtained from officially slaughtered animals. Cell culture components, including Dulbecco’s modified eagle’s medium (DMEM), Roswell Park Memorial Institute medium (RPMI 1640), Hank’s balanced salt solution (HBSS), fetal bovine serum (FBS), trypsin–EDTA, and L-glutamine were all purchased from Hyclone (Logan, UT, USA). Blood agar (sheep blood 7%), Malt Extract Broth (MEB) and MacConkey agar were supplied by LiofilChem S.R.L (Roseto d. Abruzzi, TE, Italy). The commercial antibiotics methicillin, streptomycin, and ampicillin were purchased from Fisher Scientific (Janssen Pharmaceutical, Beerse, Belgium), and the antifungal ketoconazole was from Frilabo (Porto, Portugal).

### 2.2. Extraction of Non-Anthocyanin Phenolic Compounds by Different Procedures

Three extraction methodologies were employed. Stirred maceration (MAC), a variation of classic maceration, was performed using 2 g of *Vitis vinifera* seeds and marc with 60 mL of solvent EtOH/H_2_O (80:20, *v*/*v*). The sample was kept under continuous electromagnetic stirring at room temperature for 1 h. Ultrasound-assisted extraction (UAE) was carried out using an ultrasonic device system (Sonicator QSonica, CL-33 model, Newton, CT, USA) equipped with a titanium probe. Aliquots of 3 g of dried grape seeds and marc were separately extracted in 100 mL of EtOH/H_2_O (80:20 *v*/*v*) for 27 min at 253 W power. The microwave-assisted extraction (MAE) process was performed in a microwave system (Nuwav-Ultra, NuTech, Kolkata, India). Portions of 3 g of dried grape seeds and marc were extracted with 100 mL of ultrapure water according to the following conditions: extraction time (15 min), ramp time (7 min), and temperature (80 °C). After all the extraction procedures, all extracts were separated from the solid residue by filtration (filter paper, Ø 125 mm, CMHLAB—Barcelona, Spain) and subjected to lyophilization (Freeze Dryer Telstar LyoQuest-55—Milan, Italy) for 48 h at −55 ± 0.5 °C.

### 2.3. Chemical Characterization of Non-Anthocyanin Polyphenol-Rich Extracts

The MAC, UAE, and MAE extracts were analyzed using an HPLC system equipped with a diode array detector (DAD) and in tandem mass spectrometry (HPLC-DAD-(ESI)MS/MS, Thermo Scientific, San Jose, CA, USA). Phenolic compound separation was achieved using a Spherisorb S3 C_18_ column (4.6 × 150 mm; 3 μm, Waters, Milford, MA, USA) kept at 35 °C, under a gradient of formic acid (0.1% *v*/*v* in ultrapure water, A) and acetonitrile (B). The initial proportion of mobile phase (A:B, %) of 85:15 was kept for 5 min, then changed linearly to 80:20 in 5 min, 75:25 in 10 min, 65:35 in 10 min, and 50:50 in 10 min. Finally, it returned to the initial condition in 10 min, which was kept for an additional 10 min for column reconditioning. Chromatograms were processed at 280, 330, and 370 nm for the different classes of non-anthocyanin phenolic compounds. UV-Visible (UV-Vis) spectra were acquired in the range of 180 to 700 nm. The chromatographic system was coupled to an Orbitrap Exploris 120 mass spectrometer (MS, ThermoFinnigan, San Jose, CA, USA) equipped with an electrospray ionization (ESI) source operating in negative mode. Nitrogen served as the sheath gas (50 arbitrary units). The system was operated with a spray voltage of 2.5 kV, a source temperature of 325 °C, and a vaporizer temperature of 300 °C. For fragmentation, the HCD (higher-energy collisional dissociation) energy was normalized at 30%. Full MS and data-dependent MS/MS spectra were acquired in the range of 110 to 1800 charge-to-mass ratios (*m*/*z*). Data acquisition, processing, and interpretation were performed with Xcalibur^®^ software version 2.2 (ThermoFinnigan, San Jose, CA, USA). For compound identification, retention time (Rt) and characteristics of the UV-Visible (UV-Vis) spectra and mass spectra (deprotonated molecule ([M−H]^−^), patterns of ion fragmentation (MS^2^), and relative abundance of fragment ions) were interpreted in combination and compared with standards when available and with library (MZ Cloud^®^, MZ Vault^®^, and NIST^®^), in addition to data from the literature. Quantification was performed using external calibration curves of authentic standards listed in Section 2.1, based on DAD responses. The results of the phenolic compounds were expressed as mg per g of freeze-dried extract (mg/g dw).

### 2.4. Bioactive Properties

#### 2.4.1. Inhibition of Lipid Peroxidation by Thiobarbituric Acid Reactive Species (TBARS)

A volume of 50 mL of Tris-HCl buffer solution (20 mM; pH: 7.4) was mixed with half of the mass (25 g) of pig brain (*Sus scrofa*) and homogenized. The mixture was placed into the centrifuge at 3500 rpm for 10 min. In 48-well microplates, 200 µL of extract solutions (freeze-dried extracts resuspended in their respective extraction solvents) were added, and a serial dilution was performed [35], obtaining 8 distinct concentrations in triplicate using the extraction solvent (EtOH/H_2_O 80:20 *v*/*v* for MAC and UAE, and 100% H_2_O for MAE). The extraction solvents and Trolox were used as negative and positive controls, respectively. After the serial dilution, 100 µL of ascorbic acid (0.1 mM), 100 µL of iron sulfate (10 mM), and 100 µL of the pig brain supernatant were added to the wells. The plate was incubated (37 ± 0.5 °C, 1 h) and added to 500 µL of freshly prepared trichloroacetic acid (28%; *w/v*) and 380 µL of thiobarbituric acid (2%; *w*/*v*). After all the components were added, the plate was further incubated at 80 ± 0.5 °C for 20 min. The contents of each well were transferred to a 2 mL test tube, which was centrifuged at 3000 rpm for 5 min. The supernatant of the reaction mixture was transferred to a 96-well plate and taken for absorbance reading on a SPECTROstar Nano spectrophotometer (BMG LABTECH, Ortenberg, Germany) at 532 nm. From Equation (1), the percentage of lipid peroxidation inhibition (I) was determined.

(1) $$I\left(\%\right)=\left(\frac{A_{C}-A_{S}}{A_{C}}\right)\times 100$$

where A_C_ is the absorbance of the negative control and A_S_ is the absorbance of the sample (grape by-product extract in a given concentration). The results were expressed as effective concentration (EC_50_ in mg/mL, the extract concentration needed to inhibit lipid peroxidation by 50%), obtained from the correlation between the graph of antioxidant activity percentage against the extract concentration.

#### 2.4.2. Reducing Power Assay

The different concentrations of the extracts (0.5 mL) were mixed with equal volumes of sodium phosphate buffer (200 mM, pH 6.6, 0.5 mL) and potassium ferricyanide (1% *w/v,* 0.5 mL). The mixture was incubated at 50 °C for 20 min, and 0.5 mL of 10% (*w/v*) trichloroacetic acid was added. Then 0.8 mL of the reaction mixture was transferred to the wells of a 48-well plate, followed by the addition of water (0.8 mL) and ferric chloride (0.1% *w/v*, 0.16 mL) [35]. The absorbance of the reaction mixture was measured at 690 nm, and the percentage of inhibition of the oxidative reaction was calculated according to Equation (2) below. Trolox was used as a standard (positive control), whereas buffer served as negative control (blank assay).

(2) $$I\left(\%\right)=\left(\frac{A_{S}-A_{C}}{A_{S}}\right)\times 100$$

where A_S_ and A_C_ refer to the absorbances of the extract solution (in a given concentration) and of the control (blank) assay, respectively. The extract concentration providing the EC_50_ value (mg/mL, effective extract concentration that provides 50% of the maximum antioxidant capacity) was estimated by analyzing the relation between the oxidative inhibition and the respective extract concentration.

#### 2.4.3. DPPH Radical-Scavenging Assay

The DPPH assay was performed according to Brand-Williams et al. (1995) with some slight modifications [36]. An aliquot of 30 µL of each extract solution (freeze-dried extract resuspended in its respective extraction solvent) was transferred to a 96-well plate, in which the serial dilution of the extract was performed. In the sequence, 270 μL of DPPH^●^ methanolic solution (6 × 10^−5^ M) was added to the different dilutions of the sample’s extracts and the mixtures were incubated in the dark for 1 h at room temperature. After the incubation period, the reading was undertaken at 515 nm. Trolox was used as a positive control. The radical scavenging activity (RSA%) considers the percentage of decrease in the absorbance at 515 nm as the result of the stabilization of the DPPH^●^ by an antioxidant, estimated through the following equation:

(3) $$RSA(\%)=\left(\frac{A_{C}-A_{S}}{A_{C}}\right)\times 100$$

where A_C_ is the absorbance of the DPPH solution, and A_S_ is the absorbance of the sample (extract in a given dilutions). The results were expressed as EC_50_ values (mg/mL), calculated from the graph of RSA percentage against the extract concentration.

#### 2.4.4. Antimicrobial Activity

The grape by-product extracts were evaluated according to their antimicrobial potential, and all microorganisms were obtained from a Portuguese company (Frilabo, Portugal). For Gram-negative bacteria, we used *Enterobacter cloacae* (ATCC 49741), *Escherichia coli* (ATCC 25922), *Pseudomonas aeruginosa* (ATCC 9027), *Salmonella enterica* subsp (ATCC 13076), and *Yersinia enterocolitica* (ATCC 8610). For Gram-positive bacteria, we tested *Bacillus cereus* (ATCC 11778), *Listeria monocytogenes* (ATCC 19111), and *Staphylococcus aureus* (ATCC 204305). Finally, for fungi, we evaluated *Aspergillus fumigatus* (ATCC 204305) and *Aspergillus brasiliensis* (ATCC 16404). The incubation conditions were determined according to Pires et al. (2018) [37]. Standardized TSB was used for bacteria suspension (1.5 × 10^6^ CFU/mL) and quantified using a densitometer. Suspensions of the fungi were prepared in PBS, and Tween (0.1%) standardized at 1.0 × 10^6^ CFU/mL, quantified by counting in a Neubauer chamber. A stock solution of 20 mg/mL extract was prepared in DMSO (5%; *v*/*v*) and Tryptone Soy Broth (TSB) culture medium. In a 96-well microplate, 90 μL of the extract solution was added to 100 μL of TSB, and a serial dilution was performed. Subsequently, 10 μL of inoculum was added in each of the wells, obtaining effectively tested extract concentrations, in duplicates, of between 10 and 0.075 mg/mL. Negative controls of the extract and TSB culture medium were prepared. Ketoconazole and streptomycin, and methicillin and ampicillin, were used as positive controls for the antifungal and antibacterial activities, respectively. All work was performed with sterile materials handled in laminar flow. The results have been expressed as the minimum inhibitory concentration (MIC), minimum bactericidal concentration (MBC) and minimal fungicidal concentration (MFC), this being the concentration required to inhibit and kill bacteria and fungi, respectively, in mg/mL. For bacteria, MIC and MBC values were obtained after 24 and 48 h of incubation at 37 ± 0.5 °C, respectively, and for fungi, MIC and MFC values were obtained after 72 and 144 h of incubation at 25 ± 0.5 °C, respectively.

### 2.5. Statistical Analysis

Data were compared by analysis of variance (ANOVA) followed by Tukey’s post hoc test (α = 5%) using the Statistica 7.0 software. Regression analyses and chromatogram plotting were carried out using Origin 8.5 Software. To evaluate whether there were significant differences in the antioxidant results among the three types of extracts for either sample residue, a Student’s *t*-test (α = 0.05) was applied. This treatment was carried out using MATLAB R2023a v.9.14 (MathWorks Inc., Natick, MA, USA).

## 3. Conclusions

The biowaste generated by the grape sector represents a great loss, especially for the wine industry, regarding the phytochemical compounds present in the skin, seeds, and other discarded by-products, which could offer several benefits to human health if reused in the correct way.

All extracts obtained from grape marc and seeds presented a high concentration of phenolic compounds, regardless of the extraction method used (maceration, ultrasound, and microwave-assisted extraction). The chemical characterization of the extracts is also highlighted, in order to detail the compounds and specific chemical groups obtained in each extraction methodology.

Furthermore, the extracts were also able to present high antioxidant activity in the different assays performed (TBARS, DPPH, and reducing power), in addition to being effective in inhibiting different strains of bacteria and fungi that are considered food contaminants. Overall, maceration and ultrasound-assisted extraction were able to yield the best antioxidant and antimicrobial activities in the extracts.

Therefore, this work presents a possible use of grape marc and seeds as natural preservatives for food application, due to their high concentration of bioactive compounds, and the consequent antioxidant and antimicrobial properties of the extracts obtained through different extraction methodologies.

## Figures and Tables

**Table 1:** Chromatographic and spectroscopic characteristics and quantitative data of phenolic compounds in extracts of grape seeds and marc.

						Grape Seeds		
Peak	Rt (min)	λmax (nm)	[M−H]^−^ *m*/*z*	MS^2^ (*m*/*z*) *	Tentative Identification	Quantification (mg/g dw)		
						MAC	UAE	MAE
**1**	4.12	277	169	125 (100)	Gallic acid ^1^	0.65 ± 0.04 ^a^	0.26 ± 0.02 ^b^	0.23 ± 0.01 ^b^
**2**	4.56	280	227	143 (100)	Resveratrol ^2^	0.48 ± 0.03 ^a^	0.44 ± 0.02 ^a^	0.35 ± 0.02 ^b^
**3**	4.75	295	389	227 (100)	Resveratrol-3-*O*-glucoside ^2^	0.84 ± 0.01 ^a^	0.63 ± 0.02 ^b^	0.41 ± 0.02 ^c^
**4**	4.91	275	153	135 (100)	Protocatechuic acid ^3^	0.82 ± 0.02 ^a^	0.77 ± 0.03 ^a^	0.321 ± 0.001 ^b^
**5**	5.69	324	593	505 (14), 473 (20), 383 (21), 353 (39), 325 (8)	Apigenin-6,8-*C*-diglucoside ^4^	0.59 ± 0.01 ^c^	0.51 ± 0.03 ^c^	0.46 ± 0.02
**6**	6.16	311	1169	1017 (100), 881 (5), 729 (11), 847 (25), 891 (14), 577 (65), 289 (8)	Procyanidin trimer digallate ^1^	1.88 ± 0.06 ^a^	0.65 ± 0.01	1.3 ± 0.1 ^b^
**7**	6.52	321	1017	729 (21), 865 (12), 847 (2), 577 (100), 575 (55), 559 (6), 289 (9)	Procyanidin trimer monogallate ^1^	1.01 ± 0.06 ^a^	0.9 ± 0.1 ^a^	0.86 ± 0.04 ^b^
**8**	6.92	311	865	451 (44), 425 (59), 407 (97), 289 (65)	Procyanidin trimer ^5^	2.2 ± 0.1 ^a^	1.0 ± 0.1 ^b^	1.0 ± 0.1 ^b^
**9**	7.11	309	1153	865 (22), 713 (4), 577 (33), 575 (16), 561 (20), 289 (100)	Procyanidin tetramer isomer I ^5^	1.1 ± 0.1 ^a^	0.7 ± 0.1 ^b^	0.71 ± 0.05 ^b^
**10**	8.01	274	1439	1153 (100), 865 (32), 713 (8), 577 (33), 575 (16), 561 (50), 289 (10)	Procyanidin pentamer ^5^	7.42 ± 0.21 ^a^	5.76 ± 0.48 ^b^	2.99 ± 0.05 ^c^
**11**	10.36	280	937	467 (100), 301 (23)	Trigalloyl-HHDP-glucose ^1^	0.47 ± 0.04 ^a^	nd	nd
**12**	12.32	279	1153	865 (22), 713 (4), 577 (33), 575 (16), 561 (20), 289 (100)	Procyanidin tetramer isomer II ^5^	6.49 ± 0.32 ^a^	1.4 ± 0.03 ^c^	2.66 ± 0.1 ^b^
**13**	13.02	361	609	301 (100)	Quercetin-3-*O*-rutinoside ^6^	0.31 ± 0.03 ^a^	nd	nd
**14**	13.48	368	593	285 (100)	Kaempferol-3-*O*-rutinoside ^7^	0.33 ± 0.03 ^a^	nd	nd
**15**	13.75	328	477	301 (100)	Quercetin-3-*O*-glucoronide ^8^	0.71 ± 0.1 ^b^	0.22 ± 0.01 ^c^	0.63 ± 0.05 ^b^
**16**	14.29	315	447	285 (100)	Kaempferol-3-glucoside ^7^	0.73 ± 0.03 ^b^	0.66 ± 0.05	0.60 ± 0.04 ^c^
**17**	15.21	346	461	285 (100)	Luteolin-glucuronide ^9^	0.11 ± 0.001 ^b^	0.1 ± 0.01 ^b^	0.10 ± 0.0 1 ^b^
**18**	16.04	338	463	301 (100)	Quercetin-3-*O*-glucoside ^8^	0.62 ± 0.02 ^a^	0.58 ± 0.01 ^a^	nd
**19**	17.77	356	507	345 (100)	Syringetin-3-*O*-hexoside ^10^	0.79 ± 0.05 ^a^	nd	nd
					**Total Phenolic Acids**	1.5 ± 0.1 ^a^	1.03 ± 0.05 ^b^	0.55 ± 0.01 ^c^
					**Total Condensed Tannins**	20 ± 1 ^a^	10 ± 1 ^b^	9.5 ± 0.4 ^b^
					**Total Stilbenes**	1.32 ± 0.04 ^a^	1.07 ± 0.03 ^b^	0.75 ± 0.04 ^b^
					**Total Flavonoids**	3.6 ± 0.3 ^a^	1.6 ± 0.1 ^b^	1.3 ± 0.1 ^c^
					**Total Phenolic Compounds**	27 ± 1 ^a^	15 ± 1 ^b^	13.1 ± 0.5 ^b^
						**Grape marc**		
**Peak**	**Rt (min)**	**λmax (nm)**	**[M−H]^−^ *m*/*z***	MS^2^ (*m*/*z*)*	**Tentative Identification**	**Quantification (mg/g dw)**		
**1**	4.23	277	169	125 (100)	Gallic acid ^1^	**MAC**	**UAE**	**MAE**
**20**	4.38	276	331	169 (100), 125 (3)	Galloyl glucose ^1^	0.307 ± 0.004 ^b^	0.131 ± 0.003 ^c^	0.44 ± 0.02 ^a^
**21**	4.85	280	289	245 (100)	( + )-Catechin ^5^	1.38 ± 0.05 ^b^	0.47 ± 0.01 ^c^	2.5 ± 0.1 ^a^
**22**	4.98	282	1137	1119 (42), 1011 (59) 865 (100), 847 (51), 739 (26), 577 (46), 559 (33), 407 (26)	Proanthocyanidin tetramer ^5^	1.99 ± 0.04 ^a^	0.63 ± 0.02 ^c^	1.56 ± 0.01 ^b^
**23**	5.61	276	867	287 (63), 409 (58), 577 (100), 715 (46)	Procyanidin trimer C ^5^	nd	0.49 ± 0.01 ^a^	nd
**24**	6.17	283	863	739 (100), 713 (5), 577 (25), 575 (12), 425 (25), 287 (25)	A-type procyanidin trimer ^5^	nd	0.42 ± 0.01 ^a^	nd
**25**	6.44	280	577	451 (18), 425 (82), 407 (91), 289 (100), 287 (18)	B-Type (epi)catechin dimer ^5^	2.75 ± 0.04 ^a^	0.349 ± 0.005 ^c^	1.01 ± 0.04 ^b^
**8**	6.71	279	865	739 (8), 713 (7), 695 (20), 577 (45), 575 (8), 425 (20), 407 (30), 289 (11), 287 (25)	B-Type (epi)catechin trimer ^5^	1.17 ± 0.04 ^a^	0.55 ± 0.01 ^c^	1.3 ± 0.1 ^a^
**26**	7.25	280	289	245 (100)	(-)-Epicatechin ^5^	1.48 ± 0.01 ^a^	0.62 ± 0.01 ^c^	0.87 ± 0.04 ^b^
**9**	7.57	281	1153	865 (22), 713 (4), 577 (33), 575 (16), 561 (20), 289 (100)	Procyanidin tetramer isomer I ^5^	1.26 ± 0.03 ^a^	0.26 ± 0.01 ^c^	1.1 ± 0.03 ^a^
**10**	8.36	274	1439	1153 (100), 865 (32), 713 (8), 577 (33), 575 (16), 561 (50), 289 (10)	Procyanidin pentamer isomer I ^5^	1.53 ± 0.04 ^c^	4.3 ± 0.1 ^a^	4.22 ± 0.04 ^a^
**27**	10.36	282	1439	1153 (100), 865 (32), 713 (8), 577 (33), 575 (16), 561 (50), 289 (10)	Procyanidin pentamer isomer II ^5^	nd	0.308 ± 0.003 ^a^	nd
**12**	12.75	279	1153	865 (22), 713 (4), 577 (33), 575 (16), 561 (20), 289 (100)	Procyanidin tetramer isomer II ^5^	4.21 ± 0.04 ^a^	0.717 ± 0.004 ^c^	3.4 ± 0.1 ^b^
**28**	15.29	281	1121	865 (100), 713 (8), 577 (35), 575 (26), 289 (10)	B-type proanthocyanidin tetramer ^5^	3.23 ± 0.03 ^a^	1.47 ± 0.04 ^b^	2.44 ± 0.02 ^a^
**29**	17.47	280	1017	739 (100), 713 (8), 577 (25), 575 (5), 425 (30), 289 (11), 169 (25)	Galloylated procyanidin trimer ^1^	2.0 ± 0.1 ^a^	0.727 ± 0.002 ^b^	1.2 ± 0.1 ^b^
					**Total Phenolic Acids**	0.61 ± 0.01 ^b^	0.306 ± 0.005 ^c^	0.83 ± 0.03 ^a^
					**Total Condensed Tannins**	18.1 ± 0.3 ^a^	10.2 ± 0.2 ^b^	16.1 ± 0.4 ^a^
					**Total Flavonoids**	2.9 ± 0.1 ^a^	1.1 ± 0.02 ^b^	3.3 ± 0.1 ^a^
					**Total Phenolic Compounds**	21.6 ± 0.4 ^a^	11.6 ± 0.2 ^b^	20.3 ± 0.5 ^a^

Data (phenolic compounds in mg/g freeze-dried extract) are presented as mean ± sd. * the relative abundance of respective fragment ions are presented. nd: not identified. MAC: dynamic maceration (80% EtOH); UAE: ultrasound-assisted extraction (80% EtOH); MAE: microwave-assisted extraction (H_2_O). Superscript numbers in compound identification refer to the calibration curves (where the y axis represents the peak area while the x axis refers to compound concentration in μg/mL used for their quantification, namely, 1. gallic acid (y = 131,538x + 292,163), 2. resveratrol (y = 54,835x − 29,986), 3. protocatechuic acid (y = 214,168x + 27,102), 4. apigenin-7-*O*-glucoside (y = 10,683x − 45,794), 5. ( + )-catechin (y = 84,950x − 23,200), 6. quercetin-3-*O*-rutinoside (y = 23,794x − 46,683), 7. kaempferol-3-*O*-glucoside (y = 27,328x + 2683.3); 8. quercetin-3-*O*-glucoside (y = 28,555x + 3032.3), 9. luteolin-7-*O*-glucoside (y = 27,772x − 11,351), and 10. apigenin-7-*O*-glucoside (y = 14,957y + 14,559) curves. Different letters in each row signify significant statistical differences among samples (*p* < 0.05).

**Table 2:** Antioxidant capacity assessed using TBARS, reducing power, and DPPH methods.

		TBARS	Reducing Power	DPPH
Grape seed	MAC	0.048 ± 0.003 ^a^	0.097 ± 0.014 ^a^	0.242 ± 0.007 ^a^
	UAE	0.398 ± 0.001 ^b^	0.439 ± 0.026 ^b^	0.240 ± 0.016 ^a^
	MAE	0.255 ± 0.010 ^c^	0.561 ± 0.035 ^c^	1.448 ± 0.302 ^b^
Grape marc	MAC	0.230 ± 0.100 ^a,b^	0.221 ± 0.004 ^a^	2.789 ± 0.095 ^a^
	UAE	0.023 ± 0.001 ^a^	1.708 ± 0.074 ^b^	2.277 ± 0.048 ^b^
	MAE	0.110 ± 0.003 ^b^	2.463 ± 0.083 ^c^	0.939 ± 0.082 ^c^
Trolox		3.73 ± 1.90	0.029 ± 0.003	0.043 ± 0.002

All results were expressed in EC_50_ values (mg/mL, mean ± sd). MAC: maceration extraction; UAE: ultrasound-assisted extraction; MAE: microwave-assisted extraction. Different letters among results of a given antioxidant assay (columns) within either grape seed or marc extracts are statistically different (*p* < 0.05).

**Table 3:** Antibacterial and antifungal activities of the different phenolic-rich extracts obtained from by-products of the winery sector.

	Grape Seeds						Grape Marc						Positive Controls					
	MAC		UAE		MAE		MAC		UAE		MAE		Streptomycin		Methicillin		Ampicillin	
**Gram-negative bacteria**	**MIC**	**MBC**	**MIC**	**MBC**	**MIC**	**MBC**	**MIC**	**MBC**	**MIC**	**MBC**	**MIC**	**MBC**	**MIC**	**MBC**	**MIC**	**MBC**	**MIC**	**MBC**
*Enterobacter cloacae*	>10	>10	>10	>10	>10	>10	10	>10	10	>10	>10	>10	0.007	0.007	n.d.	n.d.	0.15	0.15
*Escherichia coli*	>10	>10	>10	>10	>10	>10	10	>10	10	>10	>10	>10	0.01	0.01	n.d.	n.d	0.15	0.15
*Pseudomonas aeruginosa*	>10	>10	>10	>10	>10	>10	10	>10	10	>10	>10	>10	0.06	0.06	n.d.	n.d.	0.63	0.63
*Salmonella enterocolitica*	10	>10	>10	>10	>10	>10	>10	>10	>10	>10	>10	>10	0.007	0.007	n.d.	n.d.	0.15	0.15
*Yersinia enterocolitica*	>10	>10	>10	>10	>10	>10	>10	>10	>10	>10	>10	>10	0.007	0.007	n.d.	n.d.	0.15	0.15
**Gram-positive bacteria**	**MIC**	**MBC**	**MIC**	**MBC**	**MIC**	**MBC**	**MIC**	**MBC**	**MIC**	**MBC**	**MIC**	**MBC**	**MIC**	**MBC**	**MIC**	**MBC**	**MIC**	**MBC**
*Bacillus cereus*	10	>10	>10	>10	>10	>10	>10	>10	>10	>10	>10	>10	0.007	0.007	n.d.	n.d.	n.d.	n.d.
*Listeria monocytogenes*	10	>10	>10	>10	>10	>10	>10	>10	>10	>10	10	>10	0.007	0.007	n.d.	n.d.	0.15	0.15
*Staphylococcus aureus*	10	>10	10	>10	>10	>10	>10	>10	>10	>10	10	>10	0.007	0.007	0.007	0.007	0.15	0.15
															**Ketoconazole**			
**Fungi**	**MIC**	**MFC**	**MIC**	**MFC**	**MIC**	**MFC**	**MIC**	**MFC**	**MIC**	**MFC**	**MIC**	**MFC**			**MIC**	**MFC**		
*Aspergillus brasiliensis*	10	>10	5	>10	10	>10	5	>10	10	>10	10	>10			0.06	0.125		
*Aspergillus fumigatus*	10	>10	10	>10	10	>10	5	>10	10	>10	10	>10			0.5	1		

n.d.: not detected activity; MAC: maceration extraction; UAE: ultrasound-assisted extraction; MAE: microwave-assisted extraction; MIC: minimal inhibitory concentration (mg/mL); MBC: minimum bactericidal concentration (mg/mL); MFC: minimal fungicidal concentration (mg/mL). For fungi, the MAC extract from grape marc showed relevant results for the inhibition of the two tested fungi (*A. brasiliensis* and *A. fumigatus*), with MIC values of 5 mg/mL. Additionally, the UAE extract of grape seeds showed inhibitory activity against *A. brasiliensis* at the same concentration. All other extracts from both grape seed and marc had MIC values of 10 mg/mL.

## Data Availability

Not applicable.
